# YAP–TEAD-driven CPA4 promotes NF2-deficient meningioma growth

**DOI:** 10.64898/2026.08.05.743013

**Published:** 2026-08-07

**Authors:** Kentaro Mineji, Katerina Petrosky, Ryosuke Otsuji, Yasuhide Makino, Yuji Kibe, Eita Uchida, Daichi Hagita, Neha Singaravelan, Yukitomo Ishi, Shigeru Yamaguchi, Long- Sheng Chang, Samantha Gadd, Rintaro Hashizume

## Abstract

Neurofibromin 2 (NF2) deficiency is a driver of meningioma and other cancers, yet transcriptional effectors that sustain NF2-deficient tumors remain poorly defined. We identify carboxypeptidase A4 (CPA4) as an effector of YAP–TEAD signaling in NF2-deficient meningioma. Transcriptomic profiling identified CPA4 as a consistently upregulated effector. Across patient cohorts and specimens, CPA4 expression was enriched in *NF2* -mutant and chromosome 22q-deleted meningiomas and associated with higher tumor grade and chromosome 1p loss. CPA4 depletion impaired proliferation, disrupted cell-cycle, DNA-replication, and DNA-repair programs, suppressed intracranial tumor growth, and prolonged survival. Integrated epigenomic and functional assays identified CPA4 as a direct YAP–TEAD transcriptional target. CPA4-high meningioma models exhibited preferential sensitivity to YAP–TEAD inhibition, while verteporfin and the clinical-stage TEAD inhibitor VT3989 reduced CPA4 expression, suppressed orthotopic tumor growth, and prolonged survival. These findings uncover a targetable YAP–TEAD–CPA4 dependency in NF2-deficient meningioma and identify CPA4 as a potential biomarker for TEAD- directed therapy.

**STATEMENT OF SIGNIFICANCE:**

CPA4 links NF2 loss to oncogenic YAP–TEAD transcription, sustains meningioma growth, and marks tumor sensitivity to pharmacologic TEAD inhibition. These findings establish CPA4 as a tumor-promoting effector and potential biomarker of an actionable pathway shared across NF2- deficient cancers.

## Full Text Availability

The license terms selected by the author(s) for this preprint version do not permit archiving in PMC. The full text is available from the preprint server.

